# Artificial Intelligence and Digital Divide in Physiotherapy Education

**DOI:** 10.7759/cureus.52617

**Published:** 2024-01-20

**Authors:** Mirella Veras, Joseph-Omer Dyer, Dahlia Kairy

**Affiliations:** 1 Health Sciences, Carleton University, Ottawa, CAN; 2 School of Rehabilitation, Université de Montréal, Montreal, CAN

**Keywords:** physiotherapy students, tele-physiotherapy, physiotherapy education, physical therapy rehabilitation, physical therapy, teaching technology, education, physiotherapy, ai, artificial intelligence

## Abstract

The potential of artificial intelligence (AI) in health care and education has become increasingly evident, promising to revolutionize how healthcare professionals deliver services and how learners engage with educational content. AI enhances individualized student learning experiences and transforms education delivery by adapting to emerging healthcare advancements. We emphasize the current need for more exploration of AI's applications in day-to-day education in physiotherapy schools. We conducted a PubMed search, revealing a significant gap in research on AI in physiotherapy education compared to medical and dental education. Knowledge gaps and varied perspectives among Canadian healthcare students, including physiotherapy students, highlight the need for targeted educational strategies and ethical considerations. We conclude with a call to bridge the digital divide in physiotherapy education, stressing the importance of integrating AI to empower students and foster innovation in physiotherapy education.

## Editorial

Artificial intelligence (AI) has gained significant attention in healthcare and healthcare professionals' education [1]. As far as education is concerned, the fact that AI is likely to be more integrated into in-service delivery means that education must consider AI's contribution to the implementation of care. In this respect, professionals will need to be trained to integrate AI better to improve the accuracy of diagnosis, treatment, and monitoring, enable data-driven decision-making, identify patterns and trends, and support evidence-based decision-making [2]. On the other hand, AI will revolutionize the way education itself is delivered, offering personalized and adaptive learning experiences and enabling educators to adapt curriculum content and training methods in response to emerging healthcare advancements [3]. Unlike the conventional teaching methods in health professionals' education, AI allows for a tailored learning experience, providing for individual student needs and emphasizing areas that require improvements. AI assists educators in designing individualized learning activities, monitoring student progress, and providing immediate feedback. These prospective changes in health education have the potential to redesign conventional teaching methodologies, highlighting the influence of AI in transforming the pedagogical approaches of healthcare professionals' education [3].

Like other healthcare disciplines, the field of physiotherapy (PT) has experienced significant growth over the past decade, thanks to technological advancements, such as telerehabilitation, wearable sensors, virtual reality, and robotic-assisted therapy. These technologies have transformed how physiotherapists assess, treat, and monitor patients [4]. Despite their integration into research and clinical practices, they remain relatively unexplored in day-to-day educational institutions worldwide. Similarly, with AI, there is a potential for transformative impact in healthcare education if we are proactive in its exploration and integration.

Indeed as AI continues to advance, as with other healthcare disciplines, AI has the potential to play an increasingly important role in PT. It is reasonable to believe that it will expand into PT assessment and interventions. As a result, PT education in the future will likely need to train learners to use AI to deliver services. Moreover, it is unclear how AI will help transform PT education, given the diverse competencies related to clinical reasoning, practical skills, and attitudes that need to be developed in physiotherapists. The crucial question is whether PT schools are adequately prepared for this imminent transformation. To address this query, we examined two databases to assess the number of articles discussing AI in PT education.

PubMed and CINAHL (Cumulative Index to Nursing and Allied Health Literature) were searched on December 6, 2023, using the keywords "physiotherapy" or "physical therapy" and "education" and "artificial intelligence." Inclusion criteria were as follows: (1) Relevance to PT education: articles included in the search must explicitly pertain to the field of PT education, ensuring a direct connection to the subject matter of interest. (2) Incorporation of AI: selected articles must address or explore the integration, implications, or applications of AI within the context of PT education. (3) Publication date: no restrictions were placed on the publication date to include a broad range of relevant studies, ensuring a comprehensive review of the literature up to the search date. The exclusion criteria were as follows: (1) Irrelevance to PT education: articles unrelated to PT education, even if they mention PT or AI, were excluded to maintain the specificity of the search. (2) Absence of AI focus: articles that did not explicitly address or investigate AI's role in PT education were excluded from consideration.

The search yielded 151 and 19 articles in PubMed and CINAHL, respectively. However, in PubMed, only one of these articles specifically examined the connection between AI and PT education; in CINAHL, none specifically focused on AI within PT education. In contrast, a similar search for medical education in PubMed generated 6,137 results, with eight out of the first 10 articles addressing the association between AI and medical education. Dental education produced 241 results, with eight relevant articles found in the initial screening of 10 results. These results highlight the paucity of research on AI in PT education, and probably the fact that the discipline is lagging behind other healthcare disciplines in this respect (Table 1). The decision to restrict our search to PubMed and CINAHL was guided by the strategic choice to focus on databases known for their comprehensive coverage of healthcare-related literature. These databases were chosen because they include most of the peer-reviewed journals specifically devoted to health sciences education, including those with the highest impact factors in the field.

**Table 1:** Results of literature search in artificial intelligence within physiotherapy, medicine, and dentistry. CINAHL: Cumulative Index to Nursing and Allied Health Literature.

Discipline	PubMed results	CINAHL	Keywords
Medicine	6,137	492,848	“Artificial intelligence” or “ai” or “a.i.” AND “Medical education”
Dentistry	241	9088	“Dentistry” AND “education” or “Dental education” AND “artificial intelligence” or “AI” or “ai” or “Learning in dentistry or competencies in dentistry” or “Dental educational technology”
Physiotherapy	151	19	“artificial intelligence” or “ai” or “a.i.” AND “physiotherapy or physical therapy or physiotherapist or physical therapist” AND “education”

A recent study investigating knowledge gaps in AI among Canadian healthcare students revealed variations in perspectives and understanding across different health-related disciplines. While Canadian healthcare students expressed cautious optimism about AI's role in their field, a significant number felt inadequately informed on the subject [5]. The nationwide survey, which included diverse healthcare professions, sheds light on knowledge gaps in AI among students and contributes to advancing health education. PT students constituted 10% (217 participants) of the study. PT, medical, and dentistry students shared positive views regarding AI's development in their respective fields [5]. However, students in PT and dentistry expressed higher concerns about AI's impact on their professions and ranked their understanding of the ethical implications of AI higher than their counterparts in other healthcare disciplines. One PT student pointed out that AI might "replace high-risk treatments." Despite concerns, both PT and dentistry students agreed that AI had the potential to "greatly improve work efficiency." These perceptions underscore the need for targeted educational strategies and ethical considerations when integrating AI into the curricula of healthcare professionals [5].

The current state of healthcare education, particularly in PT, stresses a digital divide in incorporating AI knowledge into professional programs. The potential of AI in healthcare education, as highlighted by its potential applications in personalized learning experiences, diagnostic accuracy, and treatment monitoring, is evident. However, the limited exploration of AI in educational institutions on a day-to-day basis and the paucity of research specific to PT education are noteworthy. The findings from the PubMed and CINHAL searches, which revealed only one article addressing AI in PT education compared with substantial research in medical and dental education, underline the existing gap. This digital divide has implications for preparing PT schools to navigate the imminent integration of AI into the field. The study on Canadian healthcare students further highlights the need for attention to AI education, as students express optimism but feel insufficiently informed. Addressing this gap is crucial to ensure that future physiotherapists are well-equipped to leverage the benefits of AI, understand its ethical implications, and contribute effectively to the evolving healthcare area.

Considering the potential benefits of AI in PT education highlighted in this paper, several recommendations and strategies emerge to guide PT educators in effectively integrating AI into their curricula. Firstly, it is advisable to consider incorporating specialized courses on AI applications tailored to the unique needs of PT education. This could involve collaboration with AI experts to ensure comprehensive coverage and practical activities for PT students. Establishing a supportive infrastructure and offering professional development opportunities can be pivotal to overcoming potential barriers, such as resource constraints and faculty training. Moreover, addressing ethical concerns and potential resistance to change requires a proactive approach, fostering open dialogues and providing forums for educators to share experiences and best practices. Examples of resources are the multimedia appendices “Tips to Interact With Artificial Intelligence” and “Guidelines for Equitable Use of Artificial Intelligence,” published elsewhere [1]. A crucial aspect involves exploring specific AI applications within PT education, illustrating how AI can assist in analyzing patient data, recommending personalized treatment plans, and enhancing clinical simulations. This hands-on approach not only demystifies the integration process but also empowers educators to harness the benefits of AI effectively. By adopting these recommendations, PT educators can navigate the digital divide, ensuring their readiness to embrace AI in education and ultimately contribute to the education and healthcare field's evolution.

One limitation of this study is the restricted scope of databases utilized for data collection. While the current research focused on specific databases in rehabilitation and PT, it is essential to acknowledge that including additional databases could provide a more comprehensive understanding of the gaps in the literature. Future research is encouraged to explore and incorporate other databases, facilitating a comparative analysis that may contribute to a more robust interpretation of the results. This approach could enhance the generalizability and validity of findings, offering a more complete perspective on AI in PT education.

Hence, our future research plans aim to explore beyond the training aspect and explore comprehensive educational strategies, innovative instructional methods, and robust research initiatives for integrating AI into PT education. This approach extends beyond conventional training to include a broader spectrum of methodologies, emphasizing the need to utilize AI in the educational context effectively. Our goal is to empower students with practical AI skills and foster a culture of innovation within the evolving intersection of AI and health care. This study reflects a compelling call to action, addressing the imperative to bridge the digital divide in PT education and contribute meaningfully to the ongoing advancements in AI and its applications in the education and healthcare domain.
